# Adaptive lattice breathing enabled by Cu/Mg co-doping for stable anionic redox chemistry in sodium layered oxides

**DOI:** 10.1039/d5sc09077f

**Published:** 2025-12-17

**Authors:** Ziqin Zhang, Wenji Yin, Jiming Peng, Fenghua Zheng, Qichang Pan, Hongqiang Wang, Qingyu Li, Sijiang Hu

**Affiliations:** a Guangxi Key Laboratory of Low Carbon Energy Materials, School of Chemistry and Pharmaceutical Sciences, Guangxi Normal University Guilin 541004 Guangxi PR China whq74@gxnu.edu.cn sjhu@gxnu.edu.cn; b School of Chemistry and Life Health, Guilin Normal University Guilin 541199 P.R. China pjming9912@163.com

## Abstract

Advancing the energy density of sodium-ion batteries requires layered oxide cathodes with higher specific capacity, necessitating redox chemistry beyond conventional cations. Oxygen anionic redox offers a pathway but presents inherent challenges, including irreversible structural degradation, such as Jahn–Teller distortion. Here, we report that cooperative Cu/Mg co-doping triggers an adaptive lattice respiration mechanism that concurrently suppresses structural distortion and unlocks highly reversible anionic redox. Through *in situ* spectroscopy, we visualize that this dynamic process involves the oxidation of Cu^2+^ to Jahn–Teller inactive Cu^3+^, which induces a predictable lattice distortion, while Mg^2+^ orchestrates a compensatory symmetric breathing of the oxygen framework. This respiration effectively mitigates structural strain and preserves the layered integrity. Consequently, the P3-Na_0.67_Mn_0.9_Mg_0.05_Cu_0.05_O_2_ enables a remarkable reversible capacity of 258.1 mAh g^−1^. It retains 75.3% capacity after 80 cycles at 5.0C, demonstrating that adaptive lattice respiration is a viable strategy for achieving stable anionic redox chemistry.

## Abstract 2

The cooperative Cu/Mg doping in P3-Na_0.67_Mn_0.9_Mg_0.05_Cu_0.05_O_2_ enables adaptive lattice respiration, effectively stabilizing oxygen redox and achieving a high reversible capacity of 258.1 mAh g^−1^.

## Graphical abstract

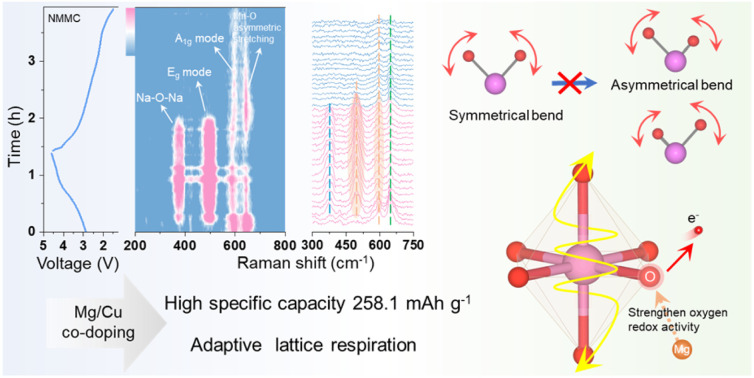

## 1 Introduction

The escalating demand for grid-scale energy storage and the geopolitical uncertainties surrounding lithium and cobalt resources have propelled sodium-ion batteries (SIBs) to the forefront of sustainable energy solutions.^1–6^ Among the various cathode candidates, sodium layered transition-metal (TM) oxides stand out for their high theoretical specific capacity and straightforward synthesis.^7–12^ However, achieving energy densities comparable to those of lithium-ion batteries remains a major challenge for SIBs.^13,14^ This pursuit necessitates a fundamental breakthrough in cathode chemistry, moving beyond the performance ceiling imposed by conventional transition-metal cationic redox reactions.^15–18^

To address this limitation, oxygen anionic redox chemistry has been identified as a viable route to access additional capacity.^5,19^ This paradigm can theoretically double the charge storage capacity compared to systems relying solely on TM redox.^20^ This is particularly crucial for manganese-based layered oxides, such as P3-type Na_0.67_MnO_2_, which are attractive for their low cost and elemental abundance but are intrinsically hampered by a limited capacity from the Mn^3+/4+^ redox couple. Nevertheless, the utility of this mechanism is hindered by its poor reversibility. The oxidation of O^2−^ ions leads to the formation of localized ligand holes, which in turn trigger oxygen loss, irreversible phase transitions, and structural degradation.^21^ A primary source of this instability is the Jahn–Teller distortion associated with Mn^3+^ (t_2g_^3^e_g_^1^) ions, which leads to severe lattice strain and capacity fade.^22^

Conventional material design strategies to mitigate these issues have largely centered on elemental doping. These approaches can be broadly categorized into two strategies, each with inherent trade-offs. The first involves the substitution with electrochemically inert or “pillar” cations, such as Mg^2+^ or Li^+^.^23,24^ These dopants can enhance the structural stability of the TM layer, suppress phase transitions, and promote oxygen redox activity by altering the local electronic structure (*e.g.*, creating Mg–O–Mn configurations with localized hole states on oxygen). However, a significant drawback of this strategy is the dilution of redox-active TM ions, which inevitably sacrifices the total capacity available from the cationic redox contribution. The second strategy employs redox-active dopants, such as Cu, which can participate in charge compensation processes. Cu is particularly interesting due to its potential for multi-electron transfer (Cu^2+^/Cu^3+^ and Cu^2+^/Cu^+^), which could theoretically compensate for the capacity loss from an inert dopant.^25^ Nevertheless, this introduces a new complexity: the Cu^2+^/Cu^3+^ redox couple is itself Jahn–Teller active. The Cu^3+^ (t_2g_^6^e_g_^2^) ion possesses a degenerate e_g_ orbital, predisposing it to a distortion that could synergize with or even exacerbate the distortion from Mn^3+^, thereby potentially negating the intended stabilizing effect.^26^ This presents a fundamental material design dilemma centered on how to simultaneously harness high capacity from both cationic and anionic redox without introducing crippling structural instability.

**Fig. 1:**
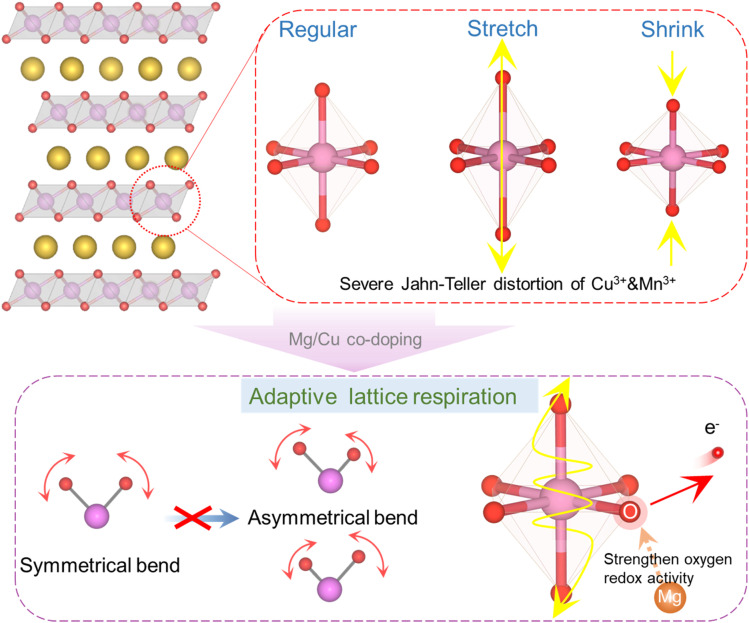
Schematic diagram of octahedral phase transformation and mechanism diagram of crystal deformation suppression by Mg/Cu co-doping.

To resolve this dilemma, we propose a co-doping strategy that reframes this apparent antagonism into a self-regulating solution (Fig. 1). We suppose that the combination of Mg and Cu in P3-Na_0.67_MnO_2_ can orchestrate an adaptive lattice breathing mechanism. In this synergistic design, Mg not only acts as a structural pillar to stabilize the oxygen framework but also electronically modulates the anionic redox. In contrast, the dynamic redox activity of Cu is harnessed to provide additional capacity. The key innovation is its management of Jahn–Teller-active Cu^2+^ ions. Instead of propagating uncontrolled distortions, the lattice responds to Cu oxidation with a localized contraction, which is offset by a Mg-induced symmetric expansion of the oxygen framework. This cooperative breathing mechanism localizes and neutralizes strain, thereby suppressing its accumulation into macroscopic phase transitions and preserving the structural integrity of the layered host during cycling.

Through *in situ* X-ray diffraction and Raman spectroscopy, we directly verify this dynamic process. The data reveal that oxidation of Cu^2+^ to Jahn–Teller inactive Cu^3+^ induces a local distortion, while Mg doping concurrently activates reversible oxygen redox and induces a compensatory lattice response. This target material exhibits a reversible capacity (258.1 mAh g^−1^), and 75.3% capacity retention after 80 cycles at 5.0C. This work demonstrates that cooperative dopant design can impart adaptive functionality, establishing a general principle for stabilizing anionic redox cathodes *via* strain-managing chemical interactions.

## 2 Results and discussion

### 2.1. Structural and morphological characterization

The inductively coupled plasma optical emission spectrometer (ICP-OES) results show that the elemental ratios of Na/Mn/Cu/Mg in all samples are in line with expectations (Table S1). The X-ray diffraction XRD patterns (Fig. S1a) show that all diffraction peaks can be indexed to a P3 phase (rhombohedral, space group symmetry of *R*3*m*). The shift of the (003) peak to a lower angle in the NMMC sample, as observed in Fig. S1b compared with NMC and NMM, reflects an expansion of the interplanar spacing caused by Mg and Cu co-doping.^27^ The XRD Rietveld refinement results (Fig. S2 and Tables S2–S5) further confirm *c*-axis expansion in NMMC. The expansion facilitates Na^+^ diffusion kinetics, thereby enhancing the rate capability. The XRD results indicate that Cu and Mg are present in the TM layers. Scanning electron microscopy (SEM) images illustrate that all samples possess a plate-like morphology of 1–2 µm (Fig. S3a, S4a and S5a). The transmission electron microscope (TEM) images reveal lattice fringes corresponding to the characteristic P3-phase arrangement (Fig. S3b, c, S4b, c, S5b and c). The lattice fringe spacing of 0.594 nm corresponds to the (003) planes (NMMC). The lattice fringe spacings of 0.556 (NMC) and 0.565 nm (NMM) correspond to the (003) planes. It further suggests that Mg and Cu doping widen the crystal plane spacing. TEM-energy dispersive spectroscopy (EDS) mappings display the uniform distribution of Na, Mn, Cu, Mg, and O elements in the lattice throughout the samples (Fig. S3d, S4d and S5d).

### 2.2. Electrochemical performance and kinetic behaviour

**Fig. 2:**
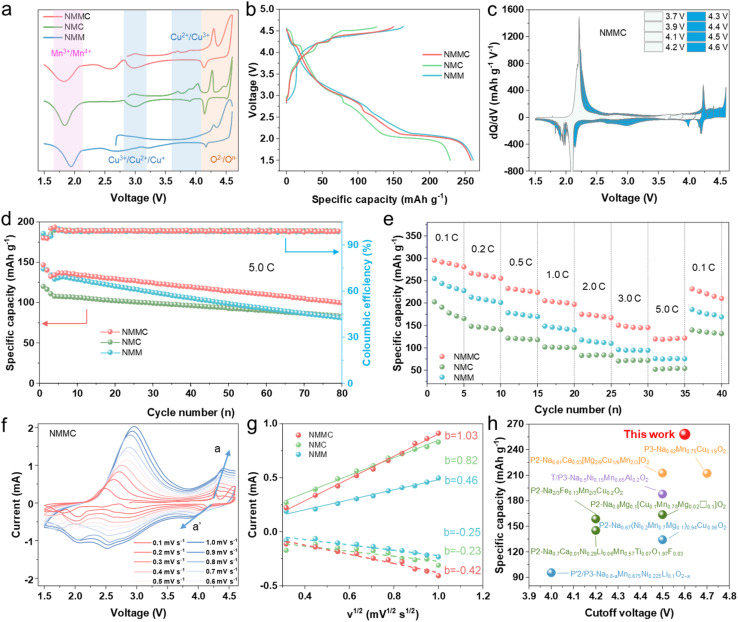
(a) CV curves of all samples within 1.5–4.6 V at a scan rate of 0.1 mV s^−1^. (b) Charge and discharge curves of all samples within 1.5–4.6 V at 0.1C. (c) The d*Q*/d*V* profiles with increasing upper cut-off voltage from 3.7 to 4.6 V at 0.1C. (d) Cycling performance of all samples within 1.5–4.6 V at 5.0C. (e) Rate capabilities of all samples. (f) Different scan rate CV curves for NMMC. (g) The relationship between the peak current (*I*_p_) and the square root of the scan rate (*ν*^1/2^) of all samples. (h) Performance comparison of layered oxide cathodes with this work.

**Fig. 3:**
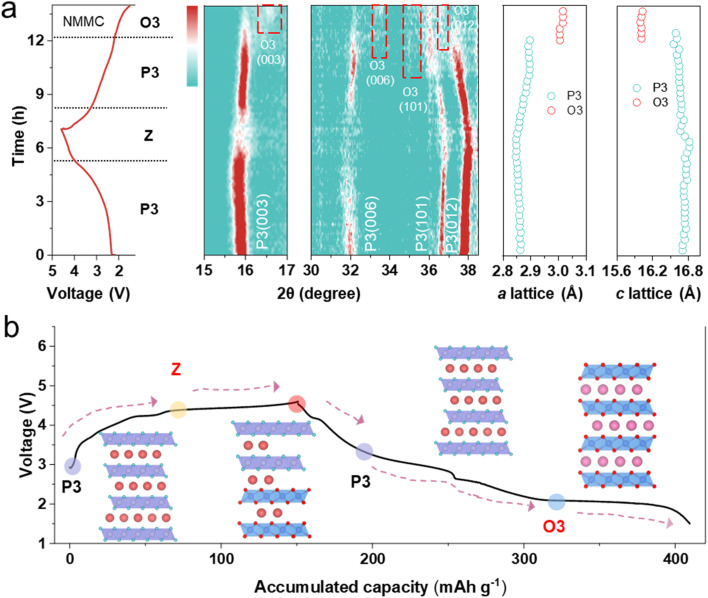
(a) *In situ* XRD patterns for the NMMC electrode alongside the corresponding charge/discharge curve on the left. (b) Schematic view of structural changes for the NMMC cathode during Na intercalation/de-intercalation.

The electrochemical performance of the NMC, NMM, and NMMC electrodes was tested by assembling half-cells. The CV curves were measured between 1.5 and 4.6 V at a scan rate of 0.1 mV s^−1^ (Fig. 2a and S6). The peak pairs at ∼2.5/1.8 V correspond to the Mn^3+^/Mn^4+^, and the peaks at 4.3–4.6 and 4.1 V correspond to O^2−^/O^*n*−^,^28^ respectively. Mg doping enhances oxygen redox activity while suppressing phase transitions at high voltages. Both NMM and NMMC exhibit smoother CV curves above 4.0 V compared to NMC. For NMC, a pair of strong peaks located at ∼4.0 V is associated with Cu^2+^/Cu^3+^, and another at 3.9 V associated with Cu^3+^/Cu^2+^/Cu^+^.^29,30^Fig. 2b shows the initial galvanostatic charge/discharge profiles of all samples at 0.1C (1C = 160 mAh g^−1^) within 1.5–4.6 V. The NMMC material delivered a first discharge specific capacity of 258.1 mAh g^−1^. This capacity is higher than that of NMC (229.1 mAh g^−11^) but slightly lower than that of NMM (261.3 mAh g^−1^), indicating that Mg doping enhances oxygen redox activity. Meanwhile, NMM and NMMC display smoother charge/discharge plateaus than NMC, consistent with the CV results.^23^ The capacity contributed by the oxygen redox reaction of NMMC was further examined by increasing the upper cut-off voltage in dQ/dV profiles between 3.7 and 4.6 V (Fig. 2c and S7). A new peak emerged at 4.4–4.6 V, which corresponded to oxygen redox reactions. During charging, charge compensation above 4.2 V arises from anionic oxidation. Within the voltage range of 2.0–4.6 V at 0.1C, the total discharge capacity contributed by anions is measured to be 69.36 mAh g^−1^. The lower voltage hysteresis of NMMC (Fig. S8a–c) suggests an outstanding reversibility during the Na^+^ extraction/insertion process. Thus, NMMC delivers a capacity retention of 75.3% after 80 cycles within 1.5–4.6 V at an ultrahigh rate of 5.0C, significantly higher than those of NMC and NMM (Fig. 2d and S8d). *In situ* XRD results (Fig. 3 and S10) revealed that Mg/Cu co-doping partially suppressed the phase transition. However, the minor phase transition still affected the cycling stability of the electrode. It may be the cause of capacity decay.

As a result, NMMC exhibits excellent rate capability from 0.1 to 5.0C (Fig. 2e) and delivers an average specific capacity of 120.3 mAh g^−1^ at 5.0C, which is much higher than the 53.4 mAh g^−1^ for NMC and 75.7 mAh g^−1^ for NMM. Upon returning to 0.1C, NMMC delivers an average discharge capacity of 220.8 mAh g^−1^, indicating that NMMC exhibits excellent structural stability and that capacity decay at high current rates originates from kinetic limitations, without incurring permanent capacity loss. The different scan rate CV curves show a steeper slope in NMMC than in NMC and NMM (Fig. 2f, g and S9), indicating significantly higher Na^+^ diffusion coefficients (6.52 × 10^−12^ cm^2^ s^−1^) in NMMC than those of NMC (4.13 × 10^−12^ cm^2^ s^−1^) and NMM (1.30 × 10^−12^ cm^2^ s^−1^). As shown in Fig. 2h and Table S6, our material demonstrates the highest specific discharge capacity among recent advances in layered oxide cathodes for sodium-ion batteries. Hence, this work provides the possibility to enhance the energy density of sodium-ion batteries.

### 2.3. Structure evolution and charge compensation mechanism

*In situ* XRD was performed to elucidate the structural evolution of the materials during cycling (Fig. 3 and S10). During charging, in all samples, as Na^+^ extraction proceeds, the electrostatic shielding effect diminishes, leading to gradual *c*-axis expansion. This corresponds to the (003) peak at ∼15.8° and the (006) peak at ∼32.0°, shifting to lower angles. Concurrently, the migration of the (101) peak at ∼36.5° and the (012) peak at ∼37.8° to higher angles indicates contraction of the *a*/*b* axes.

When the NMC electrode is charged above 2.9 V, the (003) and (006) peaks shift to higher angles, indicating the formation of the Z-phase (Fig. S10). The desodiation is coupled with the oxidation of Cu^2+^ to Cu^3+^. Cu^2+^ undergoes a tetragonal Jahn–Teller distortion, whereas Cu^3+^, with its d^8^ configuration, exhibits a more pronounced yet analogous effect due to a stronger imbalance in its e_g_ orbitals. During early desodiation, the short Cu–O bond (associated with the d_*x*^2^−*y*^2^_ orbital) contracts further, while the long Cu–O bond (related to the d_*z*^2^_ orbital) remains nearly constant, highlighting the more extreme distortion in the Cu^2+^ state.^31^ This elongates the Cu^2+^ L_6_ octahedron and leads to significant distortion within the transition-metal layer, as evidenced by the weakened intensity of the (006) diffraction peak in the *in situ* XRD pattern (Fig. S11). In contrast, no phase transition is observed in NMM throughout the entire charging process, while NMMC shows Z-phase formation only above 4.4 V (Fig. 3a). Mg^2+^ possesses a lower positive charge compared to Mn^4+^ and Cu^3+^, affecting electron delocalization within the transition-metal layer. This leads to electron redistribution toward Mg^2+^, forming MgO_6_ octahedra with localized negative charge.^32^ These octahedra act as structural “pillars”, thereby suppressing the Jahn–Teller distortion of Cu^3+^ and effectively inhibiting interlayer slip. During discharge, all materials exhibit trends opposite to those observed during charging. Cu^2+^ exhibits stronger covalency than Mn; it is more resilient to Jahn–Teller distortion.^26^ This effect is known to suppress the Jahn–Teller effect of Mn^3+^ during discharge, with the P3–O3 phase transition^33^ occurring only below 2.0 V. The O3 phase emerges in NMM below 3.0 V, whereas in NMMC, it appears below 2.1 V.

The phase transition reaction mechanism of NMMC is illustrated in Fig. 3b. During charging, the changes in the *a*- and *c*-axes of NMMC are minimal. Collectively, Mg/Cu dual doping enhances the reversibility of oxygen redox reactions and effectively suppresses the Jahn–Teller effect, inhibiting phase transitions at both high and low voltages. Consequently, NMMC maintains excellent structural stability throughout the entire charge–discharge process.

*In situ* Raman spectroscopy is sensitive to the behavioural changes of transition metals and oxygen. Therefore, *in situ* Raman spectroscopy is employed to monitor changes in TM–O and O–O bond vibrations during the charge–discharge process. All samples exhibit four initial peaks at 384, 484, 594, and 640 cm^−1^ (Fig. S12 and Table S7). The Raman peak at 384 cm^−1^ is assigned to the E_2g_ mode related to sodium variation.^34^ The peaks centered near 484 and 594 cm^−1^ are ascribed to the typical E_g_ mode (O–TM–O bending vibration) and the A_1g_ modes (TM–O stretching vibrations), respectively.^35^ Furthermore, the peaks at 610 and 640 cm^−1^ are associated with asymmetric stretching of the Mn–O bond.

**Fig. 4:**
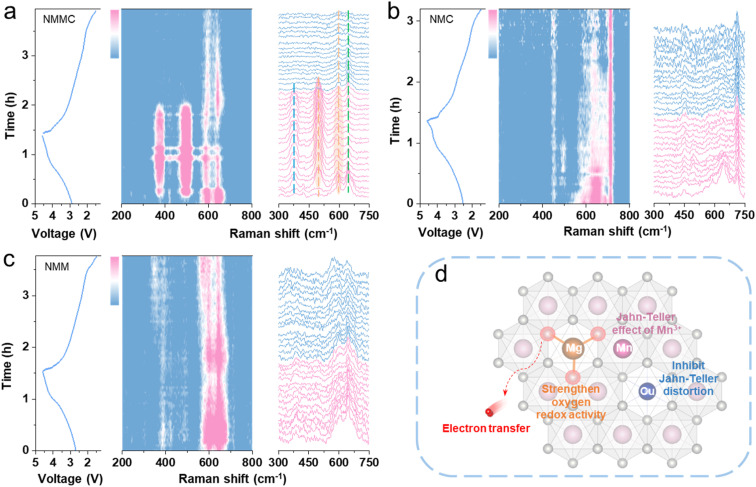
*In situ* Raman spectra for (a) NMMC, (b) NMC, and (c) NMM alongside the corresponding charge/discharge curves on the left. (d) Schematic diagram of the effect of co-doping with Mg/Cu.

In NMMC, the *a*/*b*-axes contracted, resulting in the peak at 484 cm^−1^ shifting to 481 cm^−1^ during charging (Fig. 4a).^36^ The peak at 594 cm^−1^ shifts to 585 cm^−1^ due to the extraction of Na^+^, which weakens the electrostatic repulsion between the adjacent transition-metal layers. *In situ* XRD analysis revealed that only the Cu-doped sample exhibited peak weakness of the (006) plane during charging, indicating the influence of Cu doping on the oxygen layer. The expansion or contraction of the TMO_6_ octahedra due to the Jahn–Teller effect of trivalent copper enhanced the typical E_g_ mode (O–TM–O bending vibration), resulting in the appearance of a peak at 484 cm^−1^ in Fig. 4a. Moreover, a comparison of the voltage range where the (006) plane disappears in *in situ* XRD patterns, where a Raman peak appear at 384 cm^−1^, reveals that they are identical. Therefore, the appearance of the new peak (384 cm^−1^) is associated with the distortion of the oxygen layer. Concurrently, Mg doping enhances oxygen redox activity, thereby improving the local symmetry of the O–Na–O configuration. This symmetry enhancement intensifies both the symmetric stretching vibration and the E_2g_ mode of the O–Na–O bonds, giving rise to a strong peak in the *in situ* Raman spectra (384 cm^−1^). The observed Raman signatures correspond to the collective, symmetric stretching (*i.e.*, adaptive “breathing”) of the oxygen sublattice. This cooperative breathing response effectively counteracts the local Jahn–Teller distortion induced by the transition‑metal octahedra. Notably, these spectral features disappear reversibly upon discharge, concomitant with the reduction of Cu^3+^ back to its divalent state.

In NMC (Fig. 4b) and NMM (Fig. 4c), the emergence of a new peak around 484 cm^−1^ was observed during the charging process, but no new peak appeared near 382 cm^−1^. Furthermore, the repulsion in the transition-metal layer increases upon Na^+^ removal, causing a change in structural symmetry.^37,38^ Thus, in all samples, the peaks at 594 and 640 cm^−1^ almost disappeared upon charging to high voltage. Based on *in situ* XRD results, the NMM undergoes no phase transition during charging, whereas both NMC and NMMC exhibit a P3–Z phase transition at high voltages. During discharge, the Raman peaks located at 594 and 640 cm^−1^ reappeared in both NMM and NMMC, demonstrating the reversibility of the reaction. In contrast, NMC exhibited lower reversibility, arising from structural degradation induced by the Jahn–Teller effect during discharge. Notably, these NMMC peaks revert to their original positions during discharge, indicating highly reversible structural evolution and suggesting the absence of substantial irreversible electrochemical reactions throughout cycling (Fig. 4d).

**Fig. 5:**
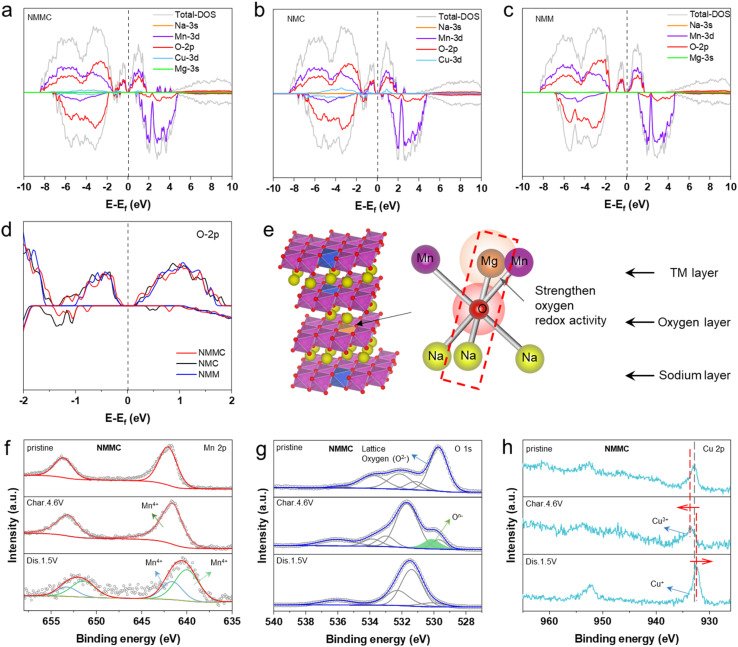
PDOS of (a) NMMC (b) NMC, and (c) NMM. (d) O 2p orbitals of all samples. (e) Left: structure of NMMC. Right: coordination around oxygen in which oxygen is coordinated octahedrally by two Mn and one Mg from the TM layer and three Na from the AM-ion layer. (f) Mn 2p, (g) O 1s, and (h) Cu 2p *ex situ* XPS spectra for NMMC.

Density functional theory was used to elucidate the redox mechanism, and the projected density of states (PDOS) for pristine samples was calculated. As shown in Fig. 5a–c, the PDOS exhibits electronic states near the Fermi level in all samples, which are the Mn 3s and O 2p orbitals. The gradual shift of the O 2p orbital toward the Fermi level is observed with Mg doping (Fig. 5d). Generally speaking, a higher O 2p state indicates that oxygen is more inclined to be oxidized upon the desodiation.^23,39,40^ Previous studies have established that Mg^2+^ in the transition-metal layer activates oxygen redox reactions. In this mechanism, the O 2p orbital hybridizes with one Mg^2+^ and two Mn^4+^ ions (Fig. 5e). The high-energy Mg 3s and O 2p orbital interaction forms a weak ionic Mg–O bond, consequently raising the O 2p state energy and ensuring its activity remains within the stability window of the electrolyte.^41^ In NMC and NMMC, Cu 3d orbitals occupied the electronic states near the Fermi level. Additionally, the PDOS structures of NMMC and NMC exhibit smaller bandgap values of ∼0.33 and 0.36 than that of NMM (∼0.43 eV).

Based on the characteristics of the charge/discharge curves, XPS measurements were applied at three different states: the pristine state, after charging to 4.6 V, and after discharging to 1.5 V. As illustrated in Fig. 5f, S14a and S15a, the Mn 2p spectra of the three pristine cathode materials present a peak located at 641.88 eV, which corresponds to Mn^4+^. When charged to 4.6 V, the valence state of Mn remains unchanged. These invariant valence states of Mn across various conditions indicate the absence of redox activity, which aligns well with the theoretically predicted electronic structures. After discharging to 1.5 V, a new Mn 2p peak appeared at 640.08 eV in all samples, suggesting a partial reduction of Mn^4+^ to Mn^3+^. The Jahn–Teller effect of Mn^3+^ induces lattice distortion, which is correlated with the P3–O3 phase transition observed using *in situ* XRD as the depth of discharge increases. Meanwhile, in the O 1s spectra of the three samples (Fig. 5g, S14b and S15b), the peaks at 529.8 eV are attributed to lattice oxygen. Upon charging to 4.6 V, all three samples exhibit a peak at 530.5 eV, attributed to (O_2_)^*n*−^.^42,43^ NMMC and NMC (Fig. 5h, S14c and S15c) reveal a dominant peak at 933.18 eV in the Cu 2p region, corresponding to Cu^2+^. When charging to 4.6 V, the binding energy of Cu 2p shifts to a higher binding energy, confirming the oxidation of Cu^2+^ to Cu^3+^. After discharging to 1.5 V, the Cu 2p peak in NMMC and NMC shifted to a lower binding energy, suggesting the partial reduction of Cu^3+^ to Cu^+^.

## 3 Conclusion

We reveal a dynamic interplay between Jahn–Teller distortions and oxygen redox reactions that establishes an equilibrium state, converting two destabilizing factors into lattice stabilization. During charging, both Cu and O can act as electron donors and undergo oxidation. When this occurs simultaneously within a single TMO_6_ octahedron, it creates an opportunity to mitigate the strong Jahn–Teller distortion of Cu^2+^. Crucially, this possibility relies on the enhanced reversibility of oxygen redox enabled by the Mg dopant. Such a dynamic equilibrium between Jahn–Teller effects and oxygen oxidation drives the evolution of non-phase-transition vibrational modes in TMO_6_ octahedra. Consequently, *in situ* Raman spectroscopy reveals two emergent vibration modes during charging, while *in situ* XRD confirms single-phase solid-solution behaviour. Furthermore, we demonstrate that Mg doping fails to effectively suppress Jahn–Teller distortions in trivalent Mn, whereas Cu^2+^ stabilizes the structure at low voltages. Ultimately, the material achieves enhanced structural stability across all charge–discharge plateaus. This target material exhibits a reversible capacity of 258.1 mAh g^−1^ with 75.3% capacity retention after 80 cycles at 5.0C. This work provides a rational design pathway for sodium-ion battery cathodes with high energy density and exceptional stability.

## Author contributions

Ziqin Zhang: methodology, experiment, data curation, data analysis, writing – original draft. Wenji Yin: experiment, data analysis, manuscript optimization. Jiming Peng: formal analysis. Fenghua Zheng: formal analysis. Qichang Pan: formal analysis. Hongqiang Wang: supervision, resources. Qingyu Li: supervision, resources. Sijiang Hu: supervision, funding acquisition, resources, writing – review & editing.

## Conflicts of interest

There are no conflicts to declare.

## Supplementary Material

SC-OLF-D5SC09077F-s001

## Data Availability

The data supporting this article are included in the supplementary information (SI). Supplementary information: experimental details, characterization data, computer method, additional figures and tables. See DOI: https://doi.org/10.1039/d5sc09077f.
