# Supplementary material for: Adaptive lattice breathing enabled by Cu/Mg co-doping for stable anionic redox chemistry in sodium layered oxides
Source: Chem Sci. 2025 Dec 17;17(7):3819–27. doi: 10.1039/d5sc09077f (PMC12766739; doi:10.1039/d5sc09077f)
Supplement: SC-017-D5SC09077F-s001 [file SC-017-D5SC09077F-s001.pdf]

Supplementary Information (SI) for Chemical Science.
This journal is © The Royal Society of Chemistry 2025

Supporting Information

Adaptive lattice breathing enabled by Cu/Mg co-doping for stable anionic redox chemistry in sodium layered oxides

Ziqin Zhang[1], Wenji Yin[1], Jiming Peng[2,], Fenghua Zheng[1], Qichang Pan[1], Hongqiang Wang[1,*], Qingyu Li[1], Sijiang Hu[1,*]*

[1]Guangxi Key Laboratory of Low Carbon Energy Materials, School of Chemistry and Pharmaceutical Sciences, Guangxi Normal University, Guilin 541004, Guangxi, PR China

[2]School of Chemistry and Life Health, Guilin Normal University, Guilin 541199, P.R. China

*Corresponding Authors: pjming9912@163.com (J.M. Peng); whq74@gxnu.edu.cn (H.Q. Wang); sjhu@gxnu.edu.cn (S.J. Hu)

1. Experimental section

1.1. Material preparation:

$Na_{0.67}Mn_{0.9}Mg_{0.1}O_2$, $Na_{0.67}Mn_{0.9}Cu_{0.1}O_2$, and $Na_{0.67}Mn_{0.9}Mg_{0.05}Cu_{0.05}O_2$ were synthesized via a conventional solid-state reaction method. Stoichiometric $NaNO_3$, $MnCO_3$, CuO, and MgO were weighed and mixed, followed by ball milling at 300 rpm for 12 h. After thorough drying, the precursors were sintered at 700 °C for 12 h under a flowing oxygen atmosphere. After cooling to room temperature and the transferred to an argon-filled glove box for storage.

1.2. Material characterization:

The molar ratios of Na, Mn, Cu, and Mg in the three materials were determined using inductively coupled plasma optical emission spectrometer (ICP-OES). Particle morphology and elemental distribution were characterized by scanning electron microscopy (SEM) and Transmission electron microscope (TEM). The crystal structure was analyzed by powder X-ray diffraction (XRD) with Cu Kα radiation with 40 kV and 40 mA over a 2θ range of 10° to 80°. The structure evolution was analyzed by *in-situ* XRD and *in-situ* Raman. X-ray photoelectron spectroscopy (XPS) was used to analyze the valence state change of all elements. Cyclic voltammetry (CV) was tested in the voltage range 1.5-4.6 V with the scan rate of 0.1 mV s^{-1}.

1.3. Electrochemical measurement:

Electrochemical measurements were conducted using a CR2032 coin cell. The cathode electrodes were fabricated with a mass ratio of active material: Super P: poly(vinylidene fluoride) = 8:1:1. The CR2032 coin cells were assembled in a glove box filled with pure argon atmosphere ($H_2O \leq 0.1$ ppm, $O_2 \leq 0.1$ ppm). Metallic sodium was employed as the counter electrode, a 1 M solution of $NaClO_4$ in propylene carbonate with 5% fluoroethylene carbonate was utilized as the electrolyte, and Whatman glass fiber (GFM) was used as the separator.

1.4. Computer methods:

All density functional theory (DFT) calculations were carried out using the Vienna Ab initio Simulation Package (VASP) [1, 2] within the generalized gradient approximation (GGA) as parameterized by Perdew-Burke-Ernzerhof (PBE) [3]. The projected

augmented wave (PAW) method[4, 5] was employed to describe the ionic cores, with valence electrons represented by a plane-wave basis set using a kinetic energy cutoff of 450 eV. Partial occupancies of the Kohn−Sham orbitals were permitted with the Gaussian smearing method (a width of 0.05 eV). For the optimization of both geometry and lattice size, Brillouin zone integrations were performed using 3×2×1 Γ-centered k-point sampling[6]. For density of states calculations, 5×3×3 Γ-centered k-point sampling was adopted. The self-consistent calculations were converged to within an energy threshold of 10^{-5} eV. The equilibrium geometries and lattice constants were adjusted to maximize the stress on each atom with a limit of 0.02 eV $Å^{-1}$. The weak interaction was described using the DFT+D3 method with an empirical correction according to Grimme's scheme[7, 8]. The spin polarization method was adopted to describe the magnetic system. To consider the strong correlation effects of the transition metals in the structure, structural optimizations and electronic structure calculations were conducted using the spin-dependent GGA plus Hubbard correction U method. The effective Ueff parameters were 3.8 eV for Mn atom and 4.0 eV for the Cu atom[9].

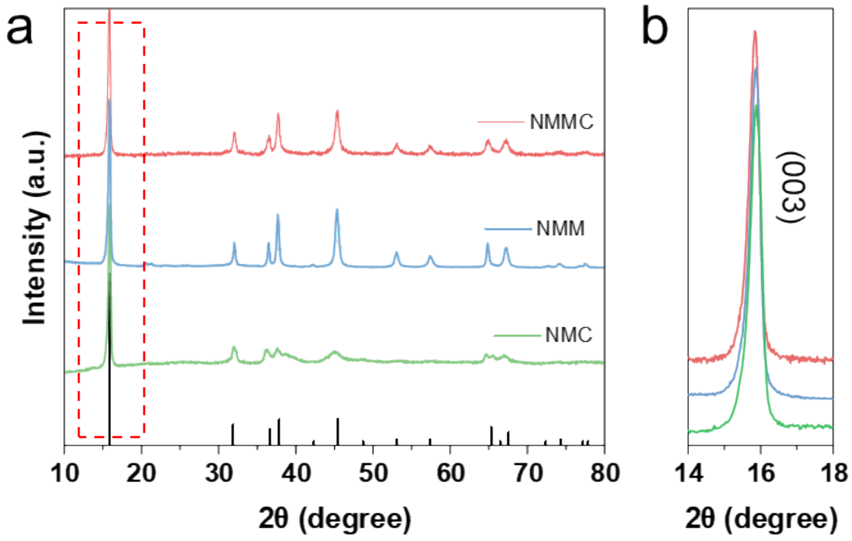

Figure S1. XRD patterns of (a) NMMC, (b) NMC and (c) NMM.

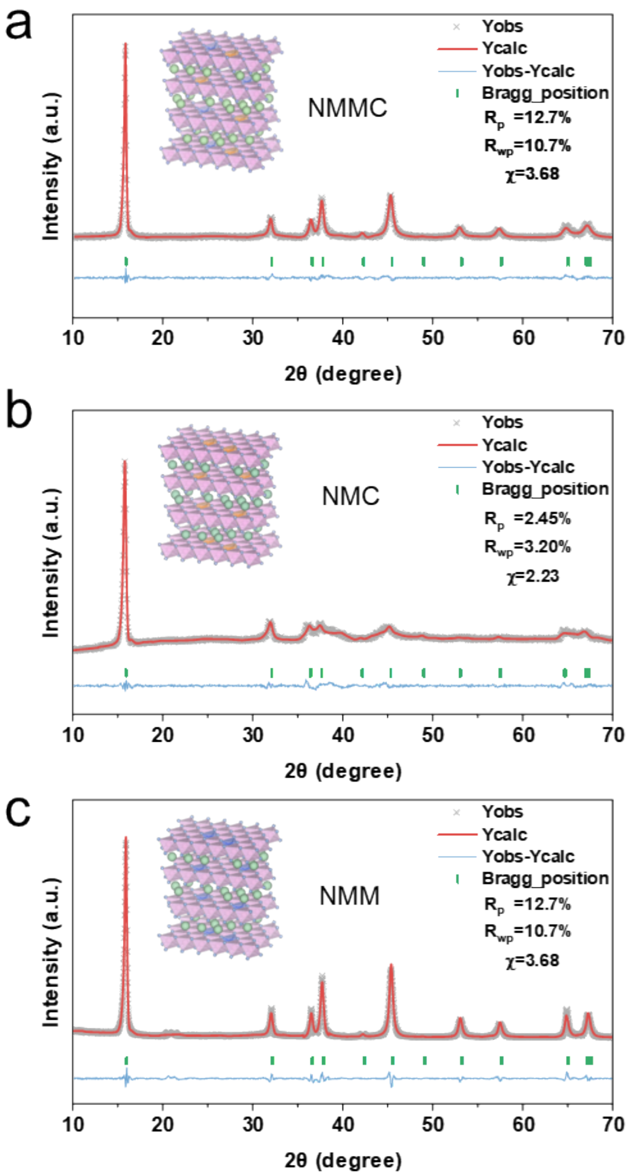

Figure S2. Rietveld refinement result of (a) NMMC, (b) NMC and (c) NMM.

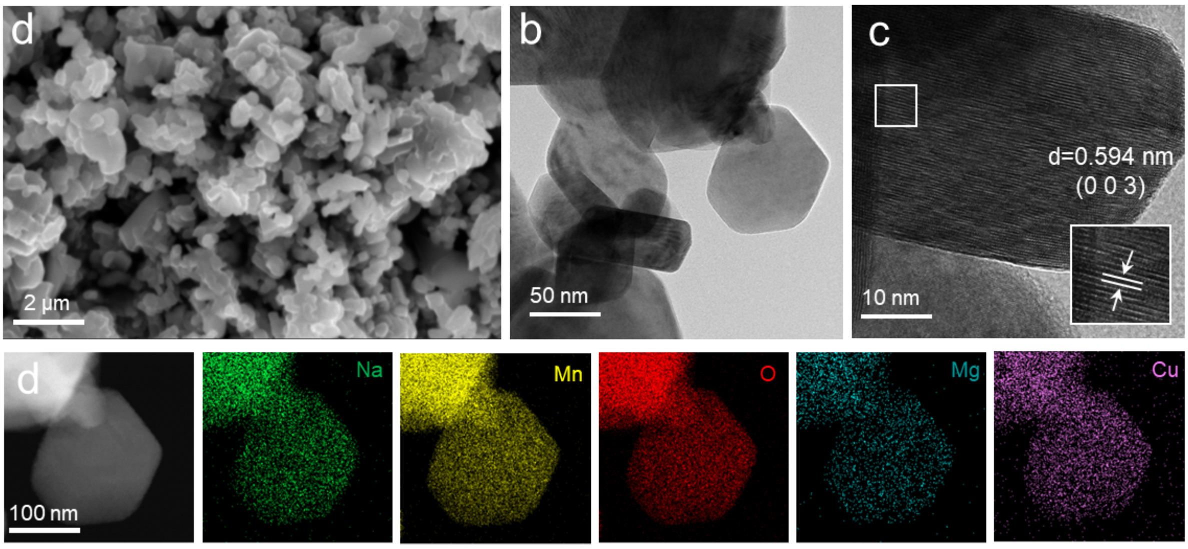

Figure S3. (a) SEM images, (b) and (c) TEM images (d) TEM-EDS mappings of NMMC.

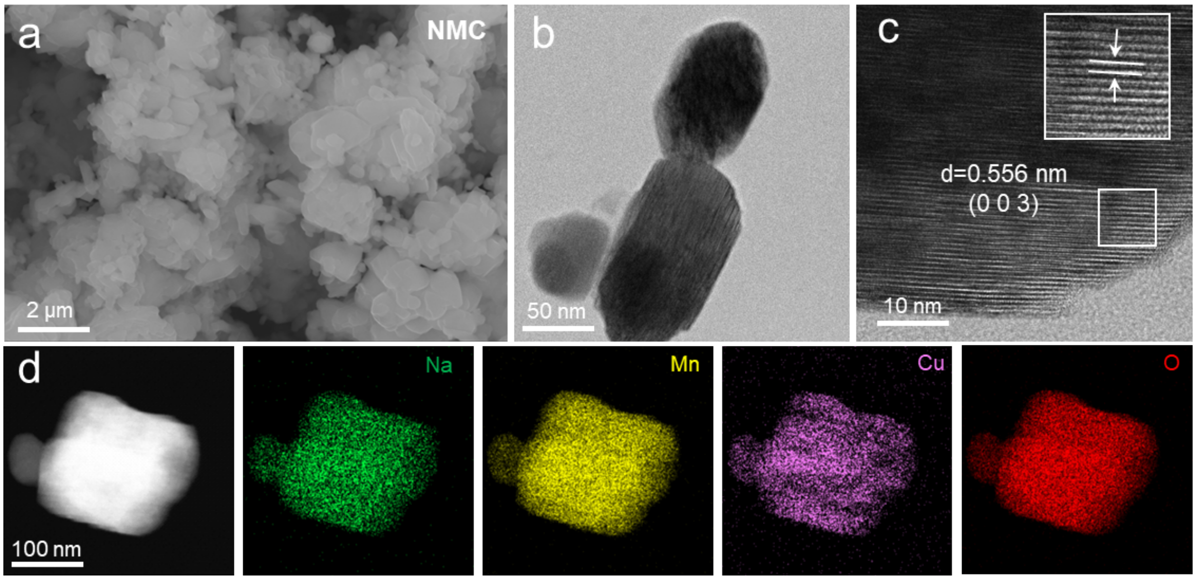

Figure S4. (a) SEM images, (b) and (c) TEM images (d) TEM-EDS mappings of NMC.

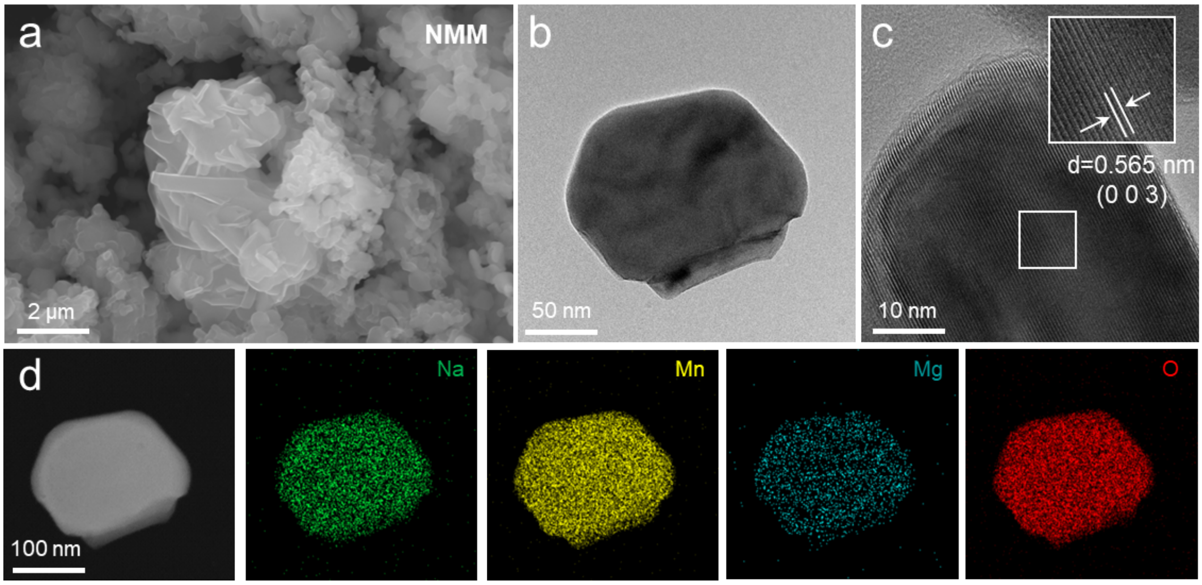

Figure S5. (a) SEM images, (b) and (c) TEM images (d) TEM-EDS mappings of NMM.

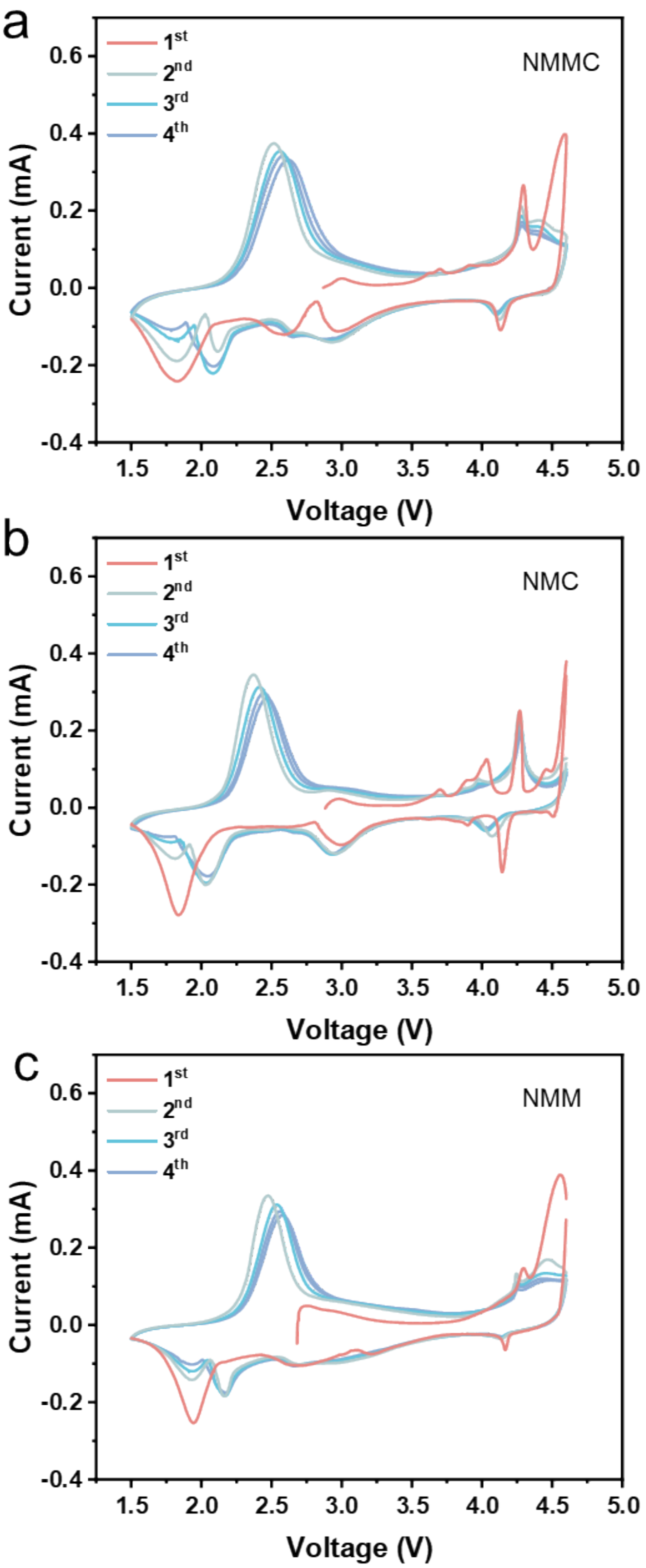

Figure S6. CV curves of (a) NMMC, (b) NMC, and (c) NMM.

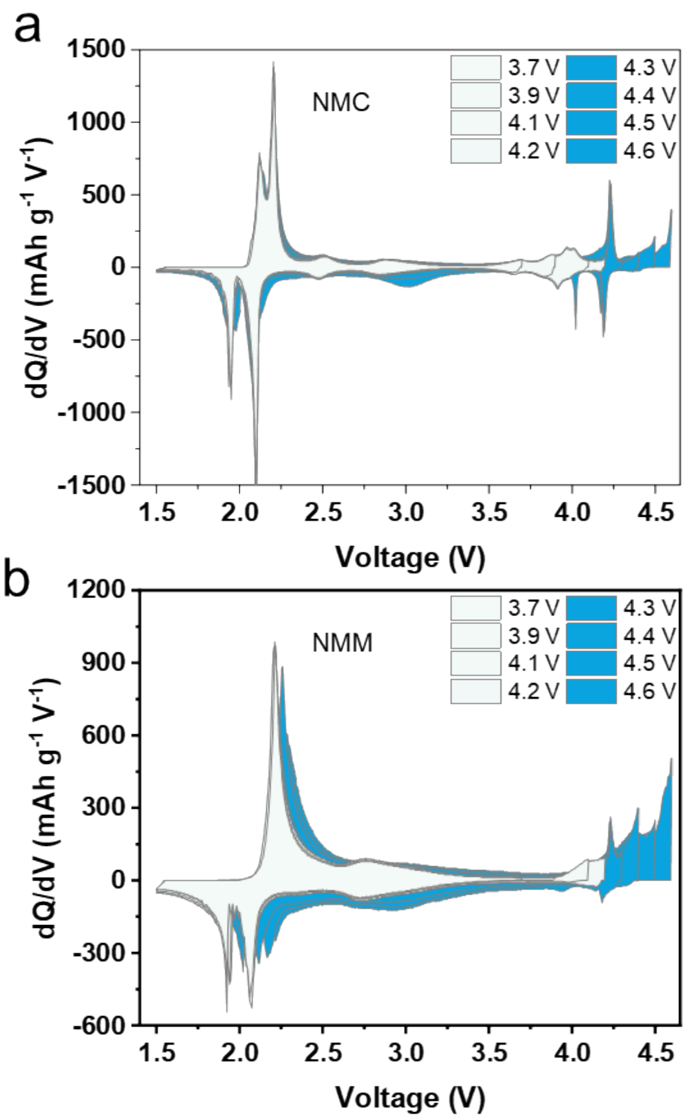

Figure S7. The dQ/dV profiles with increasing the upper cut-off voltage from 3.7 to 4.6 V at 0.1 C of (a) NMC and (b) NMM.

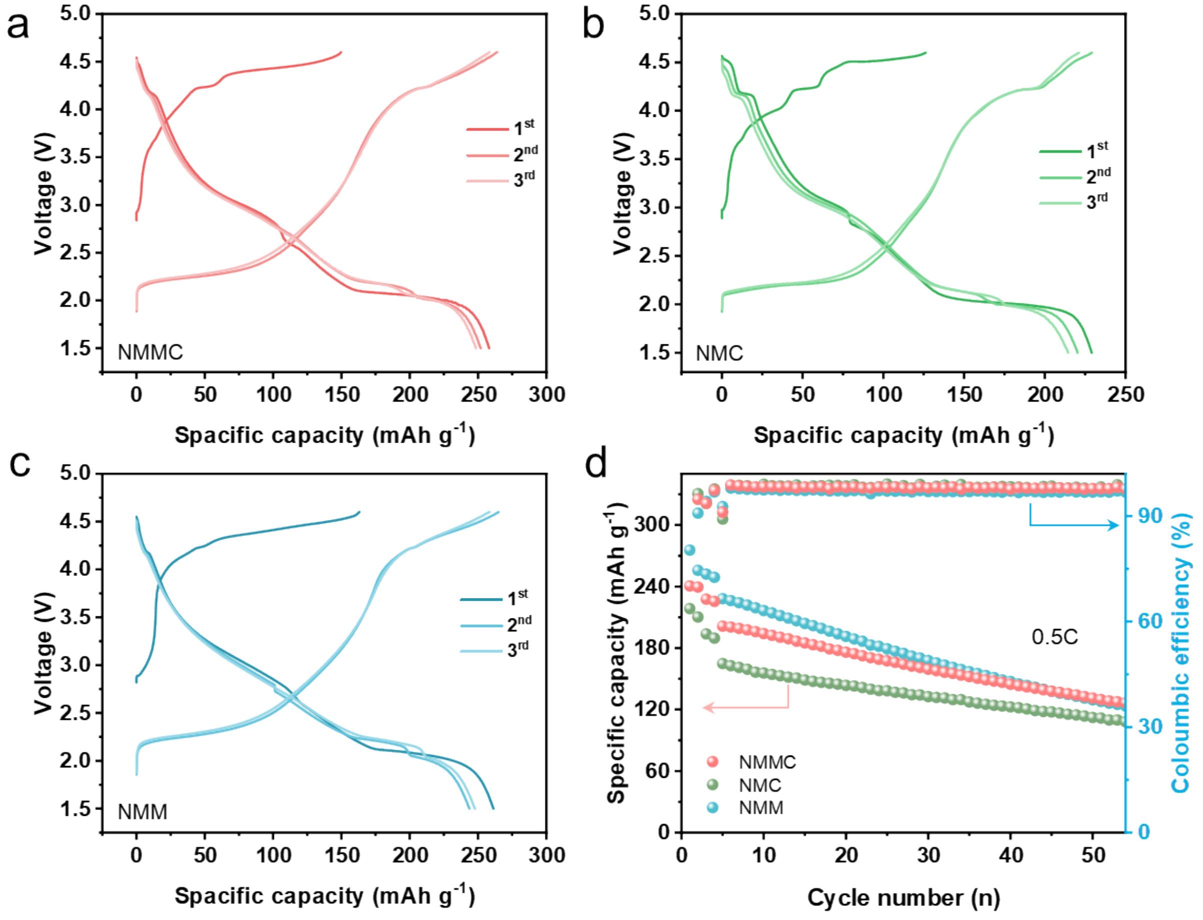

Figure S8. The first 3 cycles galvanostatic charge and discharge profiles of (a) NMMC, (b) NMC, and (c) NMM within 1.5-4.6 V at 0.1 C. (d) Cycling performance of NMMC, NMC, and NMM within 1.5-4.6 V at 0.5 C.

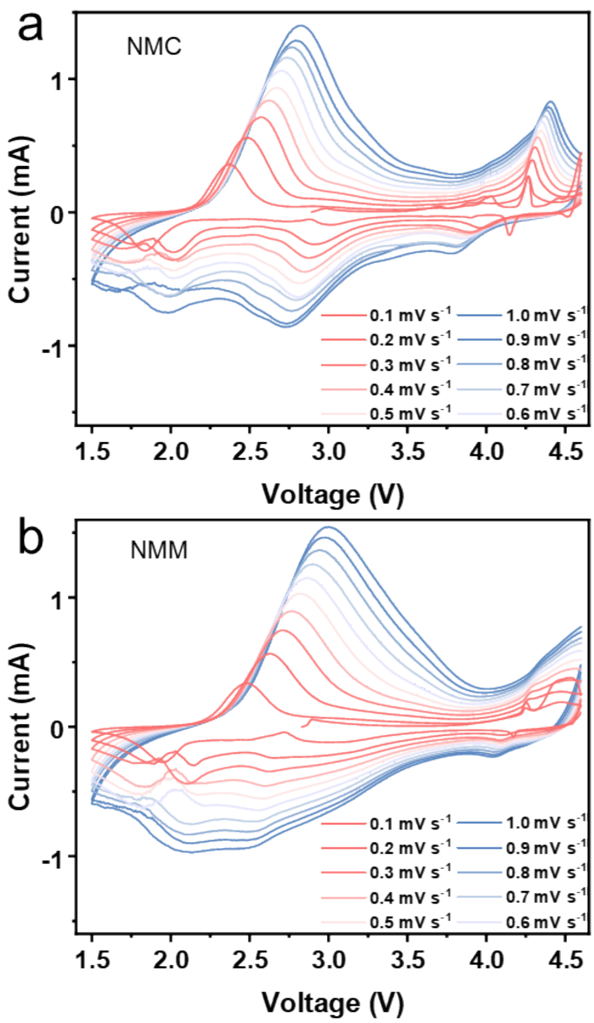

Figure S9. The different scan rates CV curves of (a) NMC and (b) NMM.

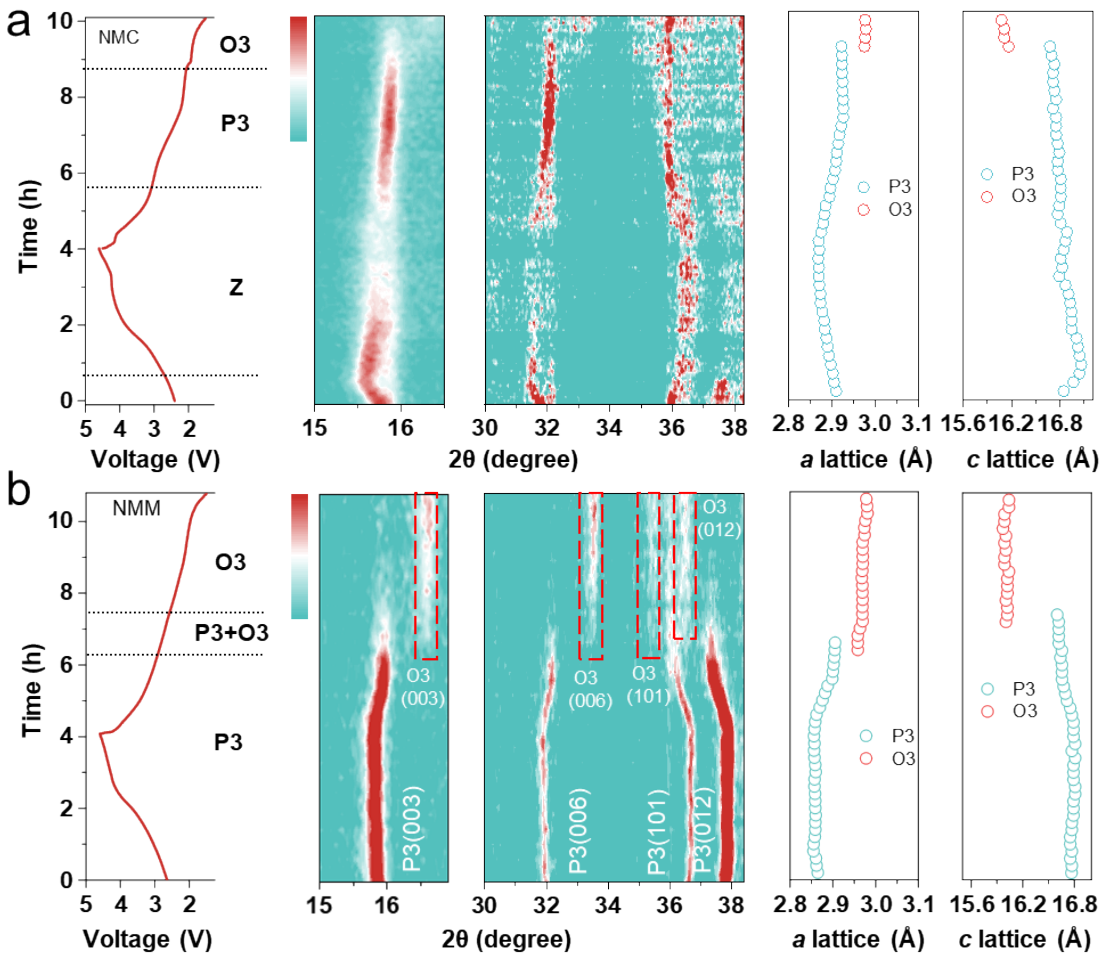

Figure S10. *In-situ* XRD patterns of (a) NMC and (b) NMM electrode alongside the corresponding charge/discharge curve on the left.

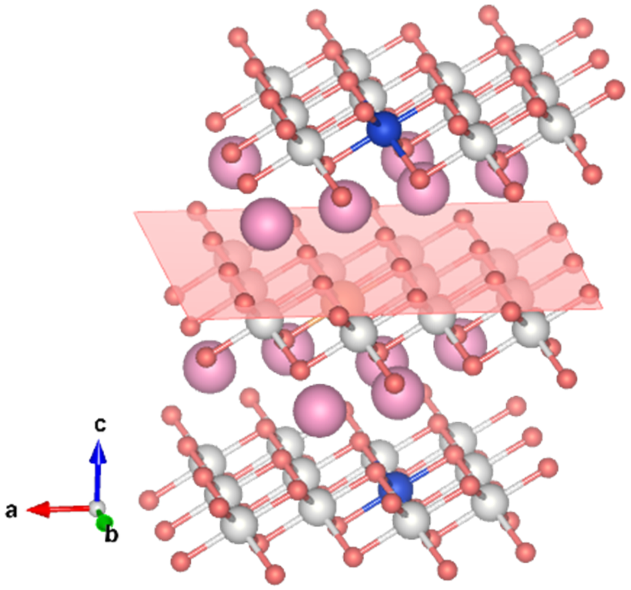

Figure S11. Crystallographic plane of (006).

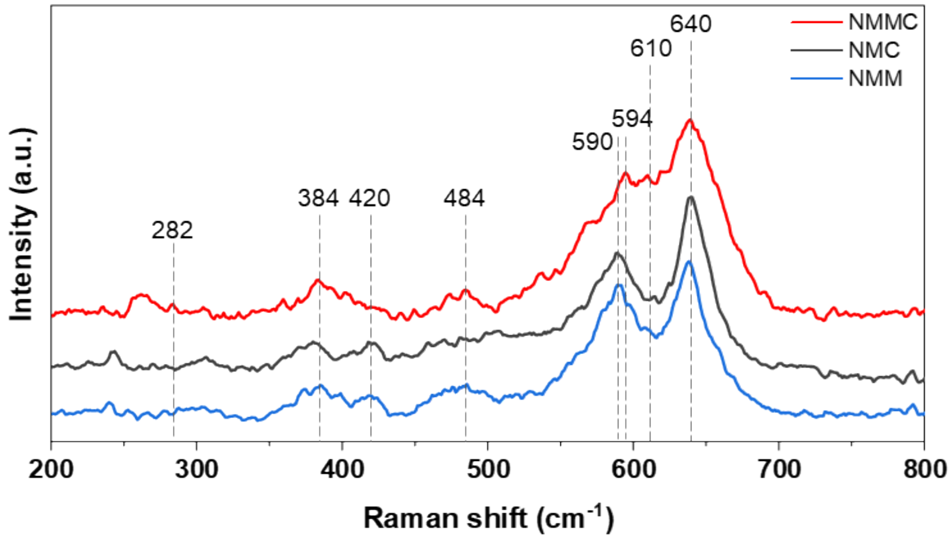

Figure S12. Raman spectra of NMMC, NMC, and NMM.

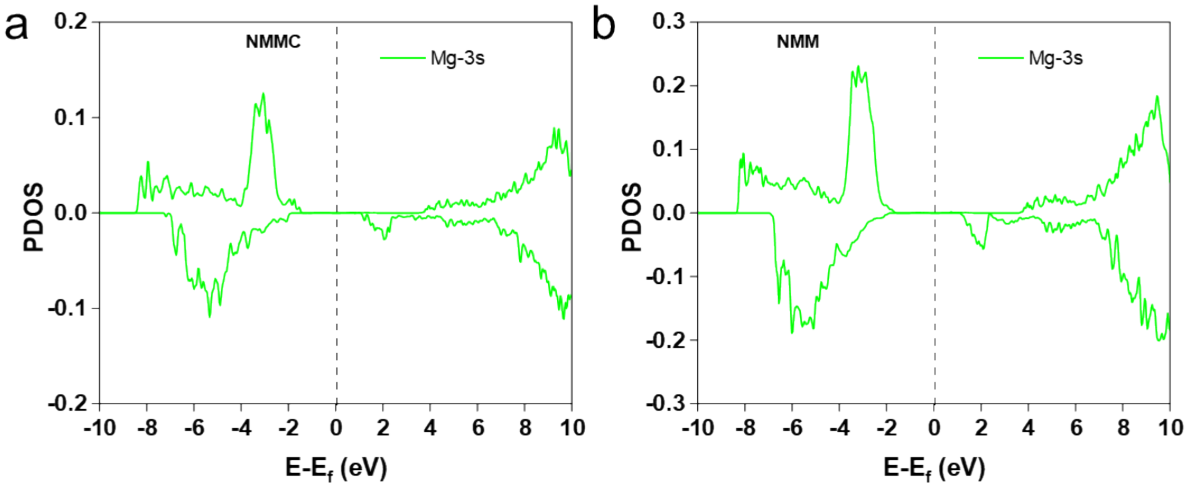

Figure S13. PDOS of Mg 3s for (a) NMMC and (b) NMM.

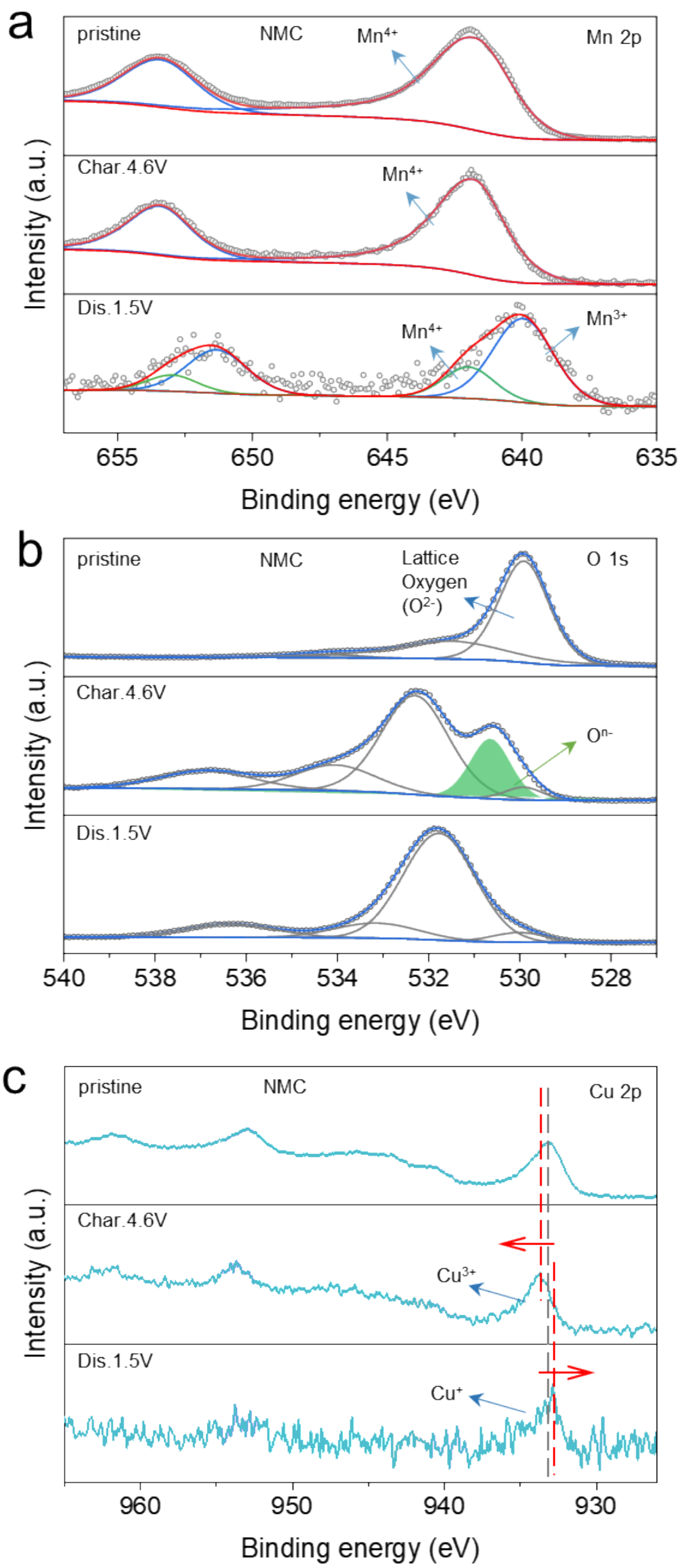

Figure S14. XPS spectra (a) Mn 2p, (b) O 1s, and (c) Cu 2p for NMC.

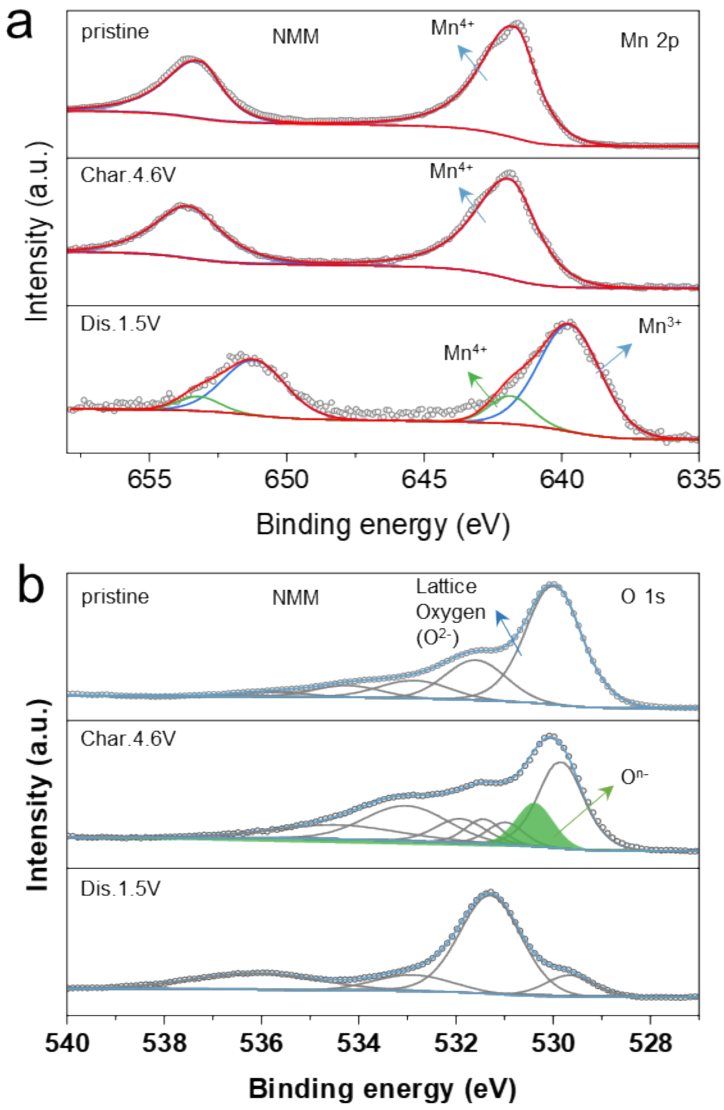

Figure S15. XPS spectra (a) Mn 2p and (b) O 1s for NMM.

Table S1. ICP-OES results of NMMC, NMC, and NMM.

Molar ratio of elements	Na	Mn	Mg	Cu
NMMC	0.66	0.89	0.06	0.05
NMC	0.64	0.89	—	0.12
NMM	0.65	0.91	0.11	—

Table S2. Refined cell parameters of NMMC, NMC, and NMM

Space group/*R*3*m*	NMMC	NMC	NMM
a (Å)	2.875	2.889	2.873
b (Å)	2.875	2.889	2.873
c (Å)	16.783	16.781	16.755
α (°)	90	90	90
β (°)	90	90	90
γ (°)	120	120	120
Volume (Å^3)	120.145	121.308	119.779

Table S3. Crystallographic data of NMMC determined from Rietveld refinement of XRD pattern.

	Atom	x	y	z	Occ
NMMC	Na1	0.00000(0)	0.00000(0)	0.16431	0.670(0)
	Mn1	0.00000(0)	0.00000(0)	0.00000	0.900(0)
	Mg1	0.00000(0)	0.00000(0)	0.00000	0.050(0)
	Cu1	0.00000(0)	0.00000(0)	0.00000	0.050(0)
	O1	0.00000(0)	0.00000(0)	0.40152	1.000(0)
	O2	0.00000(0)	0.00000(0)	0.61733	1.000(0)

Table S4. Crystallographic data of NMC determined from Rietveld refinement of XRD pattern.

	**Atom**	**x**	**y**	**z**	**Occ**
NMC	Na1	0.00000	0.00000	0.19601	0.670(0)
	Mn1	0.00000	0.00000	0.00000(0)	0.900(0)
	Cu1	0.00000	0.00000	0.00000(0)	0.100(0)
	O1	0.00000	0.00000	0.34425	1.000(0)
	O2	0.00000	0.00000	0.59574	1.000(0)

Table S5. Crystallographic data of NMM determined from Rietveld refinement of XRD pattern.

	Atom	x	y	z	Occ
NMM	Na1	0.00000	0.00000	0.25741	0.668(0)
	Mn1	0.00000	0.00000	0.09740	0.900(0)
	Mg1	0.00000	0.00000	0.09740	0.100(0)
	O1	0.00000	0.00000	0.22574	1.000(0)
	O2	0.00000	0.00000	0.66954	1.000(0)

Table S6. Comparison of the capacity retention between this work and reported layered cathodes for SIBs.

Chemical formula	Voltage range (V)	Current density (mA g^{-1})	Specific capacity (mAh g^{-1})	Reference number
**This work** **P3-$Na_{0.67}Mn_{0.9}Mg_{0.05}Cu_{0.05}O_2$**	**1.5-4.6**	**16**	**258.1**	
P'2/P3-$Na_{0.8-a}Mn_{0.675}Ni_{0.225}Li_{0.1}O_{2-x}$	2-4.0	12	95.5	[10]
P2-$Na_{0.67}(Ni_{0.2}Mn_{0.7}Mg_{0.1})_{0.94}Cu_{0.06}O_2$	2-4.5	12	134.2	[11]
P2-$Na_{2/3}Fe_{0.13}Mn_{2/3}Cu_{0.2}O_2$	2-4.2	20	158.6	[12]
P2-$Na_{0.8}Mg_{0.1}[Cu_{0.1}Mn_{0.78}Mg_{0.02}\square_{0.1}]O_2$	1.5-4.5	10	163.7	[13]
Tunnel/P3-$Na_{0.5}Ni_{0.15}Mn_{0.65}Al_{0.2}O_2$	1.5-4.5	13	187.8	[14]
P3-$Na_{0.62}Mn_{0.75}Cu_{0.19}O_2$	1.5–4.7	20	212.2	[15]
P3-$Na_{2/3}Mg_{1/3}Mn_{2/3}O_2$	2-4.5	18.578	212.56	[16]

Table S7. Summary of the Raman bands

Wavelength (cm^{-1})	Assignment
610/640	asymmetric stretching of the Mn-O
590/594	A_{1g} modes of out-of-plane M-O stretching bands
484	E_g modes of in-plane O-M-O bending
384	E_{2g} of vibration of Na-O
305	Eg modes of Cu-O
282	Mg-O

Reference

1. J. F. G. Kresse, Efficiency of ab-initio total energy calculations for metals and semiconductors using a plane-wave basis set, *Comput. Mater. Sci.*, 1996, **6**, 15.
2. J. F. G. Kresse, Efficient iterative schemes for ab initio total-energy calculations using a plane-wave basis set, *PHYS REV B*, 1996, **54**, 11169.
3. K. B. J. P. Perdew, M. Ernzerhof, Generalized Gradient Approximation Made Simple, *Phys. Rev. Lett.*, 1996, **77**, 3865.
4. P. E. Blöchl, Projector augmented-wave method, *PHYS REV B*, 1994, **50**, 17953-17979.
5. D. J. G. Kresse, From ultrasoft pseudopotentials to the projector augmented-wave method, *PHYS REV B*, 1999, **59**, 1758.
6. H. J. Monkhorst and J. D. Pack, Special points for Brillouin-zone integrations, *PHYS REV B*, 1976, **13**, 5188-5192.
7. S. Grimme, J. Antony, S. Ehrlich and H. Krieg, A consistent and accurateab initioparametrization of density functional dispersion correction (DFT-D) for the 94 elements H-Pu, *J. Chem. Phys.*, 2010, **132**.
8. S. Grimme, S. Ehrlich and L. Goerigk, Effect of the damping function in dispersion corrected density functional theory, *J. Comput. Chem.*, 2011, **32**, 1456-1465.
9. J. Liu, W. Huang, R. Liu, J. Lang, Y. Li, T. Liu, K. Amine and H. Li, Entropy Tuning Stabilizing P2-Type Layered Cathodes for Sodium-Ion Batteries, *Adv. Funct. Mater.*, 2024, **34,** 2315437.
10. C. Li, M. Li, G. Liu, H. Zhuo, Q. Li, H. Zhang, G. Pang, X. Yang, Z. Liao, K. Wang, D. Wang, B. Xiao and D. Geng, Achieving superior cyclability pouch cells with oxygen vacancy-moderated P′2/P3 hybrid layered sodium cathode materials, *ACS Appl. Mater. Interfaces*, 2024, **16**, 46216-46225.
11. J. Luo, J. Niu, X. Wang, X. Liang, C. Peng, Y. Nong, J. Zhang, M. Li, W. Ji, X. Wang, B. Zhang, X. Yuan, X. Li, J. Zhang and J. Liang, Copper substitution enhances Ni/Mn-O hybridization for improved redox kinetics and stability in sodium-ion cathodes, *ACS Appl. Energy Mater.*, 2025, **8**, 14659-14670.

12. H. Xue, H. Qiu, P. Bai, Q. Liu, Y. Zhang and W. He, Structural regulation of P2-type sodium cathode layered oxides by cation doping strategy, *Chem. Eng. J.*, 2025, **512,** 162559.

13. Z. Shi, G. Su, H. Zhang, L. Ma, X. Ma and Z. Cao, Sodium- and transition metal-sites doping improve the oxygen redox activity for developing advanced cathode materials, *J. Power Sources*, 2025, **646,** 237277.

14. J. Guo, J. Zheng, W. Zhou, X. long, X. Zhou, W. Cha, L. Feng, Y. Hao, W. Ni, Y. Li and Y. Jiang, In situ intergrowth tunnel/P3 cathode enhancing high voltage stability toward high energy sodium-ion batteries, *Adv. Funct. Mater.*, 2025, **35,** 2500604.

15. L. Rakhymbay, Z. Zhakiyeva, J. H. Yu, A. Y. Kim, H.-G. Jung, Z. Bagindyk, Z. Bakenov, S.-T. Myung and A. Konarov, Durable Cu-doped P3-type $Na_{0.62}Mn_{0.75}Cu_{0.19}O_2$ cathodes for high-capacity sodium-ion batteries, *J. Mater. Chem. A*, 2025, **13**, 6697-6708.

16. B. Chen, Y. Xin, Y. Wang, X. Ding, C. Jiang, F. Wu and H. Gao, Modulating the oxygen redox activity of an ultra-high capacity P3 type cathode for sodium-ion batteries via beryllium introduced, *Energy Storage Mater.*, 2024, **67,** 103252.
